# Supplementary material for: Multiparametric Magnetic Resonance Imaging of Penile Cancer: A Pictorial Review
Source: Cancers (Basel). 2023 Nov 8;15(22):5324. doi: 10.3390/cancers15225324 (PMC10670261; doi:10.3390/cancers15225324)
Supplement: Supplementary file 1 [file cancers-15-05324-s001.zip › cancers-2673158-supplementary.pdf]

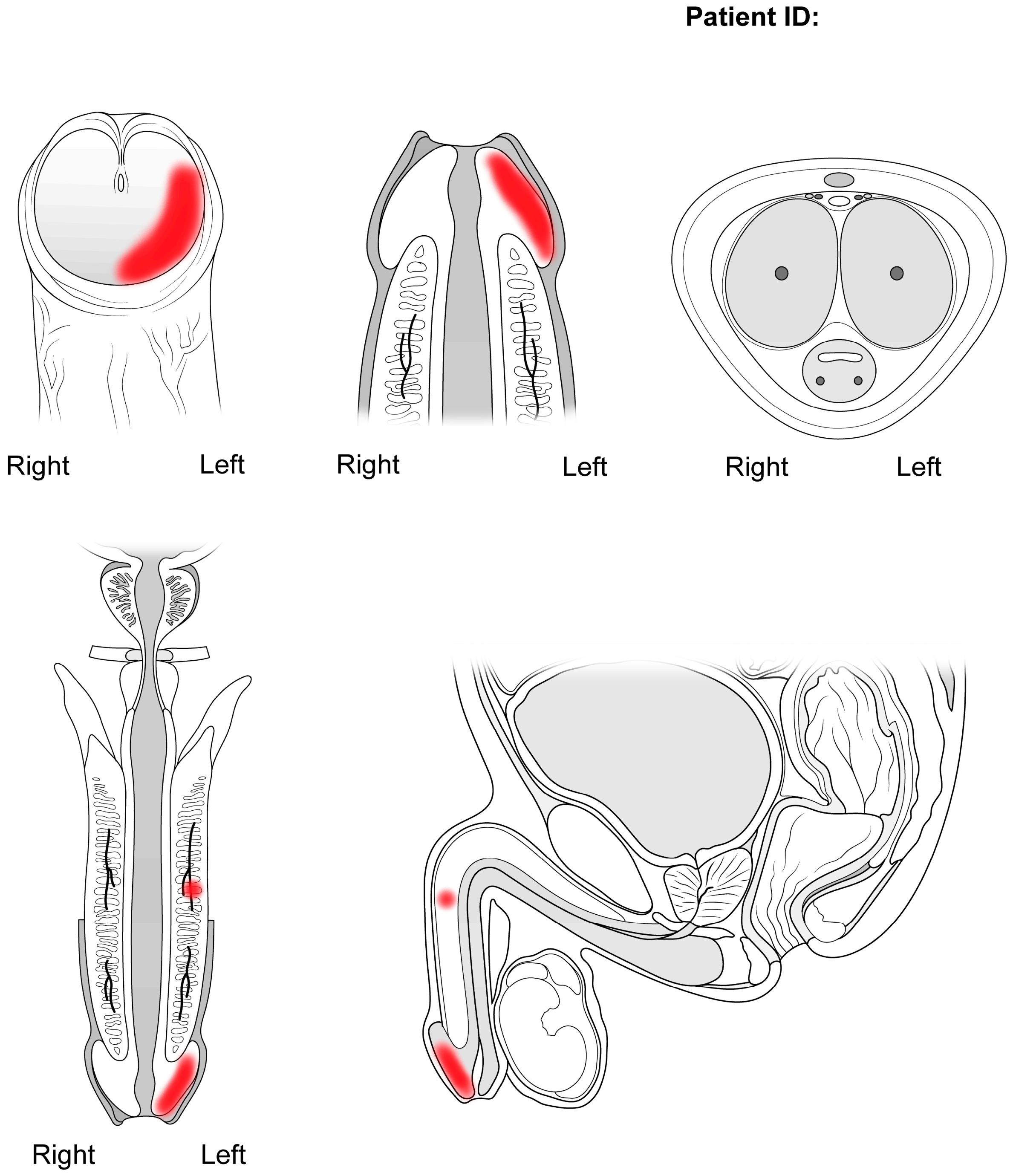

Figure S1. Example of the MRI template drawing to plot the penile cancer location.

Table S1. MpMRI protocol for the assessment of primary penile carcinoma (without DL, 3-T Vida Fit, Siemens)

MpMRI parameters	T2W	DWI RESOLVE	DWI	DCE-MRI TWIST-VIBE	T1W after Gd
Pulse sequence	2D SE	Small FOV multi-shot EPI	STIR-EPI	3D spoiled GE Dixon	3D spoiled GE Dixon
Coverage area	Primary tumor	Primary tumor	Primary tumor, inguinal and pelvic lymph nodes	Primary tumor	Primary tumor, inguinal and pelvic lymph nodes
Plane	Sag, Cor, Tra	Sag, Cor, Tra	Tra	Tra	Tra
Slice thickness (mm)					
Acquired					
Interpolated	2.0	2.3	5.0	2.2	1.2
				1.1	0.6
FOV (mm)	230	150	370	169	306
TE (ms)	99	56	64	1.35 and 2.58	2.46 and 3.69
TR (ms)	2430	2910	7190	4.4	5.8
TI (ms)	-	-	240	-	-
Flip angle	128°	180°	-	13°	12°
Gap (mm)	0.0	0.76	1.0	0.44	0.24
NEX	2	b0 = 2<br>b800 = 4	b0 = 3<br>b800 = 3	1	2
In-plane resolution (mm x mm)					
Acquired	0.72 x 0.72	1.47 x 1.47	2.5 x 2.5	1.32 x 1.32	0.82 x 0.74
Interpolated	0.36 x 0.36	0.74 x 0.74	1.25 x 1.25	0.66 x 0.66	0.37 x 0.37
ETL	17	69	120	-	-
Bandwidth (Hz/Px)	313	980	2112	810	670
Matrix					
Acquired	320 x 320	102 x 102	148 x 148	128 x 128	416 x 374
Interpolated	640 x 640	204 x 204	296 x 296	256 x 256	832 x 748
R-factor	2	2	2	4	4
b-values	-	Acquired 0 and 800, calculated 1400	Acquired 0 and 800	-	-
Time resolution (sec)	-	-	-	9.38	-
Acquisition time (min)	2:52	3:40	2:17	5:12	6:05

MpMRI - multiparametric magnetic resonance imaging, DL - deep learning, RESOLVE - readout segmentation of long variable echo-trains, TWIST - time-resolved angiography with interleaved stochastic trajectories, VIBE - volume-interpolated breath-hold examination, DWI - diffusion-weighted imaging, DCE-MRI - dynamic contrast-enhanced magnetic resonance imaging, Gd – gadolinium, SE - spin echo, FOV - field of view, EPI - echo-planar imaging, STIR - short tau inversion recovery, GE - gradient echo, Sag – sagittal, Cor – coronal, Tra – transversal, TE - echo time, TR - repetition time, TI - inversion time, NEX - number of excitations, ETL - echo train length, R-factor - reduction factor using parallel imaging acceleration.

Table S2. MpMRI protocol for the assessment of primary penile carcinoma (with DL, 3-T Vida Fit, Siemens)

MpMRI parameters	T2W DL	DWI RESOLVE	DWI	DCE-MRI TWIST-VIBE	T1W after Gd
Pulse sequence	2D SE	Small FOV multi-shot EPI	STIR-EPI	3D spoiled GE Dixon	3D spoiled GE Dixon
Coverage area	Primary tumor	Primary tumor	Primary tumor, inguinal and pelvic lymph nodes	Primary tumor	Primary tumor, inguinal and pelvic lymph nodes
Plane	Sag, Cor, Tra	Sag, Cor, Tra	Tra	Tra	Tra
Slice thickness (mm)					
Acquired					
Interpolated	2.0	2.3	5.0	2.2	1.2
				1.1	0.6
FOV (mm)	230	149	300 x 370	169	300 x 306
TE (ms)	99	55	64	1.35 and 2.58	2.46 and 3.69
TR (ms)	2620	3040	7190	4.4	5.83
TI (ms)	-	-	240	-	-
Flip angle	128°	180°	-	13°	12°
Gap (mm)	0.0	0.76	1.0	0.44	0.24
NEX	1	b0 = 2<br>b800 = 4	b0 = 3<br>b800 = 3	1	2
In-plane resolution (mm x mm)					
Acquired	0.72 x 0.72	1.47 x 1.47	2.5 x 2.5	1.32 x 1.32	0.74 x 0.82
Interpolated	0.36 x 0.36	0.74 x 0.74	1.25 x 1.25	0.66 x 0.66	0.37 x 0.37
ETL	19	69	60	-	-
Bandwidth (Hz/Px)	422	980	2111	810	668
Matrix					
Acquired	320 x 320	102 x 102	120 x 148	128 x 128	374 x 416
Interpolated	640 x 640	204 x 204	296 x 296	256 x 256	748 x 832
R-factor	4	2	2	4	4
b-values	-	Acquired 0 and 800, calculated 1400	Acquired 0 and 800	-	-
Time resolution (sec)	-	-	-	9.38	-
Acquisition time (min)	1:20	3:50	2:22	5:12	6:05

MpMRI - multiparametric magnetic resonance imaging, DL - deep learning, RESOLVE - readout segmentation of long variable echo-trains, TWIST - time-resolved angiography with interleaved stochastic trajectories, VIBE - volume-interpolated breath-hold examination, DWI - diffusion-weighted imaging, DCE-MRI - dynamic contrast-enhanced magnetic resonance imaging, Gd – gadolinium, SE - spin echo, FOV - field of view, EPI - echo-planar imaging, STIR - short tau inversion recovery, GE - gradient echo, Sag – sagittal, Cor – coronal, Tra – transversal, TE - echo time, TR - repetition time, TI - inversion time, NEX - number of excitations, ETL - echo train length, R-factor - reduction factor using parallel imaging acceleration.

Table S3. MpMRI protocol for the assessment of primary penile carcinoma (with DL, 1.5-T Sola, Siemens)

MpMRI parameters	T2W DL	DWI ZOOMit^{PRO}	DWI	DCE-MRI TWIST-VIBE	T1W after Gd
Pulse sequence	2D SE	Reduced FOV EPI	STIR-EPI	3D spoiled GE Dixon	3D spoiled GE Dixon
Coverage area	Primary tumor	Primary tumor	Primary tumor, inguinal and pelvic lymph nodes	Primary tumor	Primary tumor, inguinal and pelvic lymph nodes
Plane	Sag, Cor, Tra	Sag, Cor, Tra	Tra	Tra	Tra
Slice thickness (mm)					
Acquired					
Interpolated	2.0	2.3	5.0	2.2	1.4
				1.1	0.7
FOV (mm)	230	150	250 x 370	200	300 x 306
TE (ms)	94	71	63	2.39 and 4.77	2.39 and 4.77
TR (ms)	2820	2800	5180	6.4	6.8
TI (ms)	-	-	180	-	-
Flip angle	110°	-	-	13°	10°
Gap (mm)	0.0	0.58	0.0	0.44	0.28
NEX	2	b0 = 8<br>b800 = 16	b0 = 3<br>b800 = 6	1	2
In-plane resolution (mm x mm)					
Acquired	0.80 x 0.80	1.47 x 1.47	2.98 x 2.98	1.56 x 1.56	0.74 x 0.82
Interpolated	0.40 x 0.40	0.74 x 0.74	1.49 x 1.49	0.78 x 0.78	0.37 x 0.37
ETL	17	51	42	-	-
Bandwidth (Hz/Px)	285	1401	2122	810	670
Matrix					
Acquired	288 x 288	102 x 102	84 x 124	128 x 128	367 x 416
Interpolated	576 x 576	204 x 204	168 x 248	256 x 256	734 x 832
R-factor	4	2	2	4	4
b-values	-	Acquired 0 and 800, calculated 1400	Acquired 0 and 800	-	-
Time resolution (sec)	-	-	-	11.60	-
Acquisition time (min)	2:33	3:33	2:06	5:15	6:04

MpMRI - multiparametric magnetic resonance imaging, DL - deep learning, TWIST - time-resolved angiography with interleaved stochastic trajectories, VIBE - volume-interpolated breath-hold examination, DWI - diffusion-weighted imaging, DCE-MRI - dynamic contrast-enhanced magnetic resonance imaging, Gd – gadolinium, SE - spin echo, FOV - field of view, EPI - echo-planar imaging, STIR - short tau inversion recovery, GE - gradient echo, Sag – sagittal, Cor – coronal, Tra – transversal, TE - echo time, TR - repetition time, TI - inversion time, NEX - number of excitations, ETL - echo train length, R-factor - reduction factor with parallel imaging acceleration.
